# Vincristine impairs musculoskeletal development in pediatric mice

**DOI:** 10.1186/s12885-025-15262-x

**Published:** 2025-11-18

**Authors:** Nicholas A. Jamnick, Patrick D. Livingston, Caleb J. Gammon, Natalia M. Weinzierl, Leah J. Novinger, Douglas J. Adams, Andrea Bonetto

**Affiliations:** 1 Department of Pathology, University of Colorado Anschutz, 12800 E 19th Avenue, P18-5404C, Aurora, CO 80045 USA; 2 University of Colorado Cancer Center, Aurora, CO USA; 3 Department of Orthopedics, University of Colorado Anschutz , Aurora, CO USA; 4 Colorado Nutrition Obesity Research Center, University of Colorado Anschutz , Aurora, CO USA

**Keywords:** Chemotherapy, Vincristine, Cachexia, Muscle, Bone, Animal models, Pediatrics

## Abstract

**Background:**

Over 85% of children diagnosed with cancer now survive their disease, yet cancer and chemotherapy frequently associate with long-term health complications, including impaired musculoskeletal development. Despite high survival rates, there is limited research on how pediatric chemotherapy affects muscle and bone physiology. Vincristine, a vinca alkaloid chemotherapeutic, is widely used in pediatric oncology, but its systemic effects on the developing musculoskeletal system remain poorly understood. This study aimed to investigate the musculoskeletal consequences of vincristine in a pediatric mouse model.

**Methods:**

Four-week-old male C57BL/6J mice were administered vincristine (1.5 mg/kg, intraperitoneally, twice weekly) or vehicle for five weeks. Body mass was monitored daily. At study endpoint (day 35), skeletal muscle mass and ex vivo extensor digitorum longus (EDL) muscle function were assessed. Trabecular and cortical bone microarchitecture were evaluated via micro-computed tomography (µCT). Molecular markers of muscle atrophy and mitochondrial function were analyzed using qPCR and western blotting.

**Results:**

Vincristine-treated mice exhibited significantly reduced body mass (− 29%, *p* < 0.05), skeletal muscle mass (quadriceps − 39%, tibialis anterior − 33%, gastrocnemius − 25%, *p* < 0.05), and ex vivo EDL muscle force (− 28%, *p* < 0.05). Muscle fiber cross-sectional area was reduced (− 22%, *p* < 0.05), and SDH staining revealed a shift from oxidative to glycolytic fibers. Molecular analyses showed increased phosphorylation of STAT3^Tyr705^ (+ 267%, *p* < 0.05), no changes in the phosphorylation of AKT^Ser473^ (*p* < 0.05), elevated expression of Atrogin-1 (+ 105%, *p* < 0.05) and MUSA1 (+ 122%, *p* < 0.05), and decreased PGC-1α expression (− 44%, *p* < 0.05), overall suggesting enhanced protein degradation and mitochondrial dysfunction. The µCT analysis revealed significant trabecular bone loss (BV/TV − 84%, Tb.Th − 18%, Tb.*N* − 52%, Conn.D − 89%, *p* < 0.05) and cortical thinning (Ct.Th − 21%, *p* < 0.05). Plasma CTX-1 levels were significantly higher (+ 51%, *p* < 0.05) in the vincristine-treated mice, indicating increased bone resorption.

**Conclusions:**

Vincristine impairs musculoskeletal development in pediatric mice, leading to muscle atrophy, muscle mitochondrial dysfunction, and bone loss. Altogether, these findings underscore the need for further research into the long-term systemic effects of this frequently prescribed pediatric anticancer agent and the development of interventions to preserve musculoskeletal health in childhood cancer survivors.

**Supplementary Information:**

The online version contains supplementary material available at 10.1186/s12885-025-15262-x.

## Introduction

Over the past five decades, survival rates for childhood and adolescent cancers have improved markedly, with over 85% of patients now expected to survive to adulthood [1–4]. This progress is largely attributable to advances in treatment, surgical techniques, and access to care. However, the intensive therapies required to achieve these outcomes can lead to adverse musculoskeletal effects. While the acute impact of chemotherapy on adult skeletal muscle is well documented, little is known about the long-term effects of anticancer agents on muscle development in pediatric populations. Despite high survival rates, the molecular mechanisms underlying treatment-related growth impairments and long-term musculoskeletal complications remain poorly understood.

Chemotherapeutic agents target rapidly dividing cells, but their lack of specificity also damages healthy tissues, leading to side effects such as nausea, anorexia, anemia, and muscle weakness [5]. Preclinical studies, including our own, have shown that chemotherapy can induce musculoskeletal changes resembling cancer-associated cachexia [6, 7]. However, most of this research has been conducted in adult mouse models (>12 weeks old), leaving the pediatric effects largely unexplored. Our group recently demonstrated that Folfiri, a chemotherapeutic commonly used in adults, causes persistent musculoskeletal defects, including muscle atrophy, reduced muscle function, reduced bone integrity, and impaired mitochondrial homeostasis in 4-week-old mice [8]. While informative on the long-term effects of anticancer agents, this model does not fully reflect pediatric treatment protocols. Therefore, in the present study, we sought to better understand the impact of vincristine, a chemotherapeutic widely used in pediatric patients with hematologic and neurologic cancers, on musculoskeletal development during early life.

Given our recent work in pediatric mouse models, it is becoming increasingly evident that early-life development is highly susceptible to disruption by cancer and chemotherapy [8, 9]. Chemotherapeutic agents such as vincristine may have profound effects on musculoskeletal development during critical growth periods. Vincristine functions by preventing proper chromosome segregation during mitosis, thereby being responsible for mitotic arrest and ultimately leading to apoptosis of the cell [10–12]. While these processes are essential for its anticancer efficacy, they may also interfere with normal cellular proliferation and differentiation processes that are vital for bone and muscle development.

Despite vincristine’s widespread use in pediatric oncology, the extent to which it impairs muscle growth, function, and cellular integrity during critical developmental windows remains unclear. Addressing this knowledge gap is essential for developing targeted interventions that preserve musculoskeletal health without compromising the therapeutic effectiveness of cancer treatment.

To investigate this, we evaluated the musculoskeletal effects of vincristine in a preclinical model of early development. Four-week-old male C57BL/6J mice were treated with vincristine or the vehicle over a five-week period. Throughout the study, body composition was monitored, and skeletal muscle tissues were collected at study endpoint for detailed morphological and molecular analyses. This work aims to clarify how vincristine impacts muscle development during growth, contributing to a more comprehensive understanding of long-term musculoskeletal outcomes in pediatric cancer survivors.

## Methods

### Cell cultures

Murine C2C12 skeletal myoblasts were grown in DMEM (10% fetal bovine serum and 1% penicillin/streptomycin) and maintained at 37 °C in a 5% CO_2_ humidified atmosphere. Myotubes were generated by exposing the myoblasts to DMEM containing 2% horse serum and replacing the medium every other day for up to 5 days. Fully differentiated C2C12 myotubes were treated with 500 pM vincristine (V8879, Millipore Sigma, St. Louis, MO, USA) for up to 48 h. The concentration of vincristine and duration of exposure was determined based on our in vitro pilot experiments, concluding that concentrations above 1 nM are toxic, whereas 500 pM induces a reduction in myotube diameter >48 h. Furthermore, our in vitro experiments were also supported by published evidence examining the influence of vincristine on skeletal muscle cells [13].

### Assessment of myotube size

To determine the effects of vincristine on myotube size, cell layers were fixed in ice-cold acetone-methanol (50/50) and incubated with an anti‐myosin heavy chain antibody (MF‐20, 1:200; Developmental Studies Hybridoma Bank, Iowa City, IA, USA) and an AlexaFluor Green 488‐labelled secondary antibody (Invitrogen, Grand Island, NY, USA). Analysis of myotube size was performed by measuring the average diameter of long, multi‐nucleated fibers (*n* = 250–350 per condition) avoiding regions of clustered nuclei on a calibrated image using the ImageJ 1.43 software [14], as also shown in our previous works [15].

### C2C12 fusion index

Images were processed in a custom Python notebook (Myofusion_automated.ipynb) that performs nuclei segmentation and enforces a myotube definition. Briefly, DAPI images were converted to a single channel (blue if RGB), background-subtracted using a low-percentile estimator (5th percentile), smoothed with a Gaussian filter (σ ≈ 1.2), thresholded (Otsu or adaptive), and cleaned with morphological operations. When enabled, touching nuclei were separated via distance-transform watershed. Size filters excluded debris and outliers (typical range ~ 30–5000 px). The fusion index (FI) was computed as the number of nuclei intersecting qualified myotubes divided by the total nuclei per image, then averaged across the four fields to yield one FI value per well. All parameters (thresholding mode, watershed, size filters, and minimum nuclei per myotube) were held constant across conditions.

### Animals

All experiments were conducted with the approval of the Institutional Animal Care and Use Committee at the University of Colorado Anschutz Medical Campus, Aurora, CO and were in compliance with the National Institutes of Health Guidelines for Use and care of Laboratory Animals. In order to investigate the effect of vincristine on musculoskeletal health in young animals, 4-week-old C57BL/6J (purchased from The Jackson Laboratory, Bar Harbor, ME, USA) male mice (*n* = 5) were treated with vincristine (1.5 mg/kg subcutaneously, twice weekly) and monitored daily. The dosage was determined through pilot studies in which animals were administered vincristine at doses ranging from 0.5 to 2.0 mg/kg. A dose of 1.5 mg/kg administered twice weekly was found to be non-lethal and did not produce adverse effects that would disrupt the treatment schedule. Control mice (*n* = 5) received an equal volume of vehicle (sterile saline) injected subcutaneously. Mice were weighed daily, then euthanized under light isoflurane anesthesia after 35 days. Tibialis anterior muscles were frozen in liquid nitrogen cooled isopentane for histological assessments. The remaining tissues were harvested and weighed, then snap frozen in liquid nitrogen and stored at − 80 °C for further studies.

###  Assessment of muscle cross sectional area (CSA)

To assess skeletal muscle atrophy, 10-µm-thick cryosections of tibialis anterior muscles taken at the mid-belly were processed for immunostaining. Briefly, sections were fixed in 4% PFA for 5 min at room temperature, washed 3 × 5 min in PBS, blocked for 1 h at room temperature and incubated overnight at 4 °C dystrophin primary antibody [1:50; #MANDRA11(8B11), Developmental Studies Hybridoma Bank, Iowa City, IA], followed by a 1 h secondary antibody (AlexaFluor 555, 1:1000, A21127, Thermo Fisher Scientific, MA, USA) incubation at room temperature. Stained sections were imaged using an Olympus APX100 automated microscope (Tokyo, Japan). CSA was quantified in QuPath version 0.4.2 [16] using cellpose extension [17]. Cellpose trained model and QuPath cellpose extension script available upon request. A total of ~ 500 fibers were traced for every tibialis muscle section.

### Succinate dehydrogenase (SDH) staining in muscle

To assess muscle oxidative capacity, 10-µm-thick cryosections of tibialis anterior muscles taken at the mid-belly were processed for succinate dehydrogenase staining (SDH). Sections were incubated for 30 min at 37 °C in a Copley jar in 50 mM Na-Succinate, 0.08 mM phenazine methosulfate. Slides were washed in deionized H_2_0, 3 × 1 min and mounted with PBS-glycerol and imaged using an Olympus APX100 microscope (Tokyo, Japan).

### Western blotting

Total protein extracts were obtained homogenizing 100 mg quadriceps muscle tissue in radioimmunoprecipitation assay (RIPA) buffer [154 mM NaCl, 1.0% NP-40, 0.25% sodium deoxycholate, 0.1% sodium dodecyl sulfate (SDS), 1 mM ethylenediaminetetraacetic acid, and 65.2 mM Tris, pH 7.4) completed with 5 mM Na_3_VO_4_, 0.4 mM NaF, 0.2 mM MG-132 Proteosome Inhibitor (NC2684146, Invivogen, Carlsbad, California, USA) 0.02 mM phenylmethylsulfonyl fluoride, and protease inhibitor cocktail tablet (Roche, Indianapolis, IN, USA] and (Thermo Scientific, Waltham, MA, USA). Cell debris were removed by centrifugation (15 min, 18,000 g), and the supernatant was collected and stored at − 80 °C. Protein concentration was determined using the bicinchoninic acid (BCA) protein assay method (Thermo Scientific, Waltham, MA, USA). Protein extracts (15–40 µg) were then electrophoresed in 4–15% gradient SDS Criterion Tris-HCl precast gels (Bio‐Rad, Hercules, CA, USA). Gels were transferred to nitrocellulose membranes (Bio‐Rad, Hercules, CA, USA). Membranes were blocked with 5% Bovine Serum Albumin (BSA) in TBS-Tween (0.1%) at room temperature for 1 h, followed by an overnight incubation with diluted antibody in Tris Buffered Saline-Tween (TBS-T) (0.1%) at 4 °C with gentle shaking. After washing with TBST-T the membrane was incubated at room temperature for 1 h with either Anti‐rabbit IgG (H + L) DyLight 800 or Anti‐mouse IgG (H + L) DyLight 600 Secondary (Cell Signaling Technologies, Danvers, MA, USA). Blots were then visualized with Odyssey Infrared Imaging System (LI‐COR Biosciences, Lincoln, NE, USA). Antibodies (from Cell Signaling Technologies) used were pSTAT3^Y705^ (#9145), STAT3 (#8768), pAKT^Ser473^ (#9271), AKT (#9272), and Ubiquitin (#3933). Optical density measurements were taken using the Image Lab Software (Bio‐Rad, Hercules, CA, USA). Quantification of protein expression was measured relative to the total protein in each lane and for phosphorylation targets was quantified relative to the total protein. Primary antibody dilution was 1:1000. Secondary antibody dilution was 1:5000.

### Real-time quantitative polymerase chain reaction (qRT-PCR)

Total RNA from quadriceps was isolated using the miRNeasy Mini kit (Qiagen, Valencia, CA, USA) and following the protocol provided by the manufacturer. RNA was quantified by using a Synergy H1 spectrophotometer (BioTek, Winooski, VT, USA). Total RNA was reverse transcribed to cDNA by using the Verso cDNA kit (Thermo Fisher Scientific, Waltham, MA, USA). Transcript levels were measured by Real‐Time PCR (7500 Fast Real-Time PCR System: Applied Biosystems, Waltham Massachusetts) using the TaqMan gene expression assay system (Life Technologies, Carlsbad, CA, USA). Expression levels for Atrogin‐1 (Mm00499523_m1), MuRF‐1 (Mm01185221_m1), MUSA1 (Mm00505343_m1), PGC-1α (Mm01208835) were detected using Taqman Probes. Gene expression was normalized to TATA‐binding protein (TBP) (Mm01277042_m1) levels using the standard 2^−ΔCT^ methods.

###  Ex vivo muscle contractility

Extensor digitorum longus muscles were subjected to whole-muscle contractility assessment, as done previously [18]. The EDLs were dissected, and stainless-steel hooks were tied to both tendons using 4 − 0 silk sutures. The muscles were placed between a mounted force transducer (Aurora Scientific Inc, Aurora, Canada) and incubated in a stimulation bath containing Tyrode solution (121 mM NaCl, 5.0 mM KCl, 1.8 mM CaCl_2_, 0.5 mM MgCl_2_, 0.4 mM NaH_2_PO_4_, 24 mM NaHCO_3_, 0.1 mM EDTA, and 5.5 mM glucose) supplemented with continuous O_2_/CO_2_ (95/5%). Force data was collected and analyzed with the Dynamic Muscle Control/Data Acquisition and Dynamic Muscle Control Data Analysis programs (Aurora Scientific Inc, Aurora, Canada) and EDL muscle weight and L0 were used to determine specific force.

### Analysis of bone microstructure

After euthanasia, the right femur was dissected from each mouse, fixed for 2 days in 10% neutral buffered formalin, and then transferred into 70% ethanol for micro computed tomography (µCT) scanning on a high-throughput µCT specimen scanner (µCT50; Scanco Medical AG, Bassersdorf, Switzerland). Femurs were imaged in 70% ethanol at 70 kVp (200µA) within a 20 mm diameter field of view, collecting 2000 conebeam projections per revolution at an integration time of 500 msec. Three-dimensional images were reconstructed at 10 μm resolution using standard convolution back projection algorithms with Shepp and Logan filtering and rendered at a discrete voxel density of 1,000,000 voxels/mm3 (isometric 10 μm voxels). Bone mineral density was calibrated to a discrete-step hydroxyapatite phantom and segmented from marrow and soft tissue in conjunction with a constrained Gaussian filter to reduce noise, applying mineral density thresholds of 550 and 700 mg HA/cm3 for trabecular and cortical bone, respectively. Volumetric regions for trabecular bone analysis were selected within the endosteal borders to include the secondary spongiosa of femoral metaphyses located ~ 1 mm (~ 7% of length) from the growth plate and extending 5% of femur length proximally. Trabecular morphometric parameters were measured without imposing a presumed structural model to obtain direct measures of trabecular volume fraction (BV/TV), thickness (Tb.Th), number (Tb.N), spacing (Tb.Sp), and connectivity density (Conn.D). Cortical morphometric parameters measured were cortical thickness (Ct.Th), cortical area (Ct.Ar), fraction of cortical bone relative to total bone (Ct.Ar/Tt.Ar; %). Polar MOI reflect the amount of bone and how it is distributed and its resistance to force.

### Enzyme-linked immunosorbent assay (ELISA)

The levels of circulating C-terminal telopeptide of type I collagen (CTX-1) were measured in the plasma from EDTA-treated blood collected from vehicle- and vincristine-treated mice using a specific ELISA kit (NB030329; Novus Biologicals Mouse CTX-1 ELISA Kit, Centennial, USA) according to the manufacturer’s protocol.

### Statistics

Statistical analyses were performed using GraphPad Prism 9.4.1 (GraphPad Software, San Diego, CA, USA). Two-tailed Student’s t-tests were employed to determine differences between control and vincristine treated hosts. A 2-way repeated-measures analysis of variance (ANOVA) was performed, followed by Bonferroni’s post hoc comparisons, for longitudinal measures and ex vivo muscle contractility of the EDL. In general, variance was tested throughout our study, and in most cases, there were no significant differences. When a variance was significant, a Welch’s test was used. Statistical significance was set at *p* < 0.05, and data were presented as mean ± standard deviation(SD), unless otherwise noted.

## Results

### Vincristine promotes myotube thinning and inhibits myoblast fusion into myotubes

**Fig. 1:**
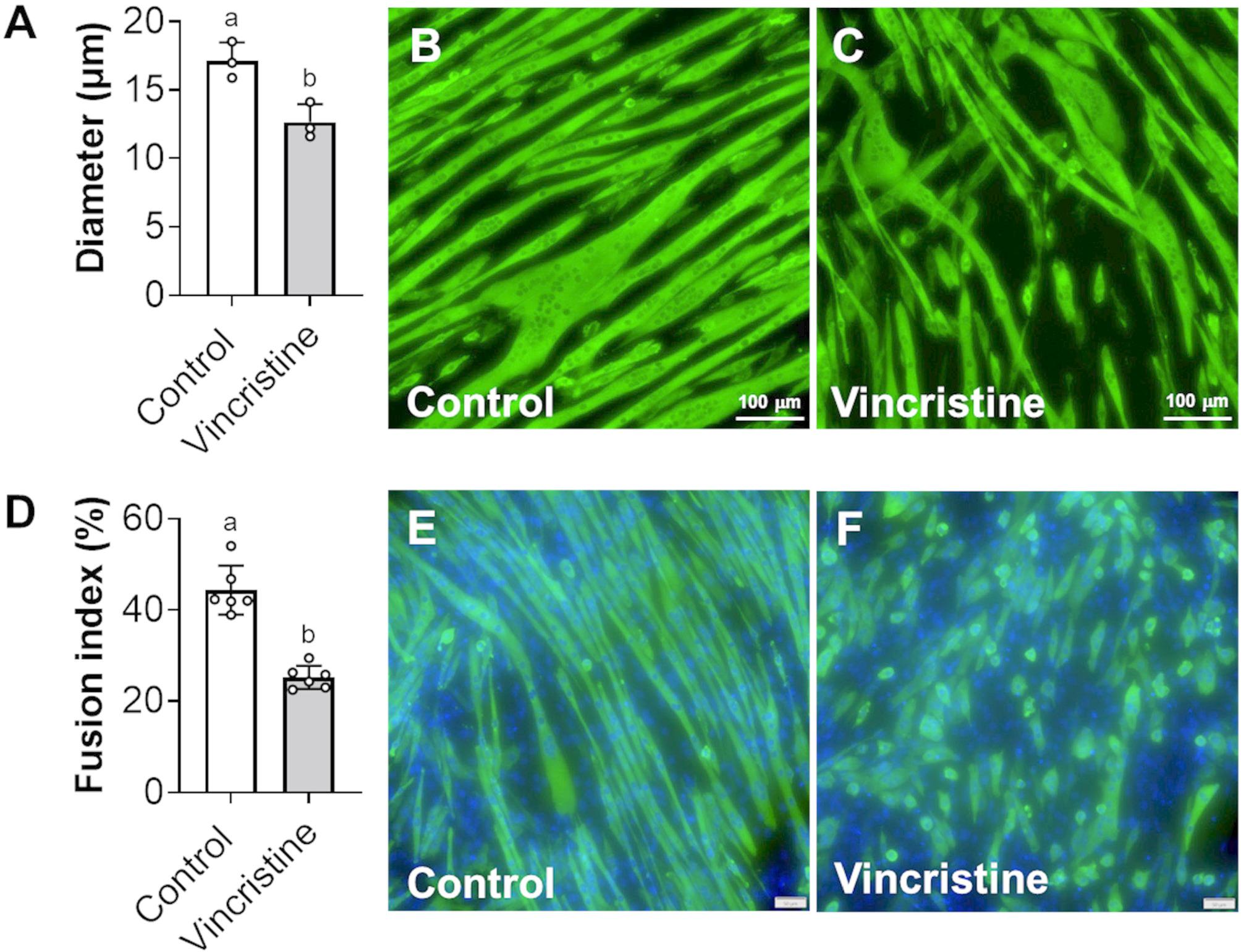
Vincristine promotes myotube atrophy. **A** Diameter of C2C12 myotubes exposed to vincristine expressed as μm. **B**, **C** Representative images of myotubes exposed to vincristine (500 pM). Magnification: 20X. Scale bar: 100 µm. **D** Fusion index of C2C12 cells. The fusion index was calculated as the percentage of total nuclei incorporated into myotubes with three or more nuclei, compared to the total number of nuclei in the field of view. **E**, **F** Representative images of myotubes exposed to vincristine (500 pM). Magnification: 20X. Scale bar: 50 µm. Data is representative of one experiment, with three technical replicates per experiment. Data expressed as means ± SD. Different letters denote significant differences: *p*<0.05

To evaluate the effect of vincristine on myotube morphology, 5-day differentiated C2C12 myotubes were treated with vincristine (500 pM) for 48 h. The treatment resulted in a significant reduction in myotube diameter compared to control cells (Control: 17.1 ± 1.3 μm, Vincristine: 12.6 ± 1.3 μm; -35%, *p* < 0.05) (Fig. 1A-C). To evaluate the effect of vincristine on myoblast differentiation, C2C12 cells were treated with vincristine (500 pM) at day 0 and day 2 during a 5-day differentiation protocol. Vincristine significantly impaired myoblast fusion into multinucleated myotubes (Control: 44.4 ± 5.4%, Vincristine: 25.2 ± 2.6%; -43%, *p* < 0.05). These findings indicate that vincristine directly impairs myotube integrity, promotes cellular atrophy and inhibits myotube formation.

### Vincristine stunts growth and changes in organ masses

**Fig. 2:**
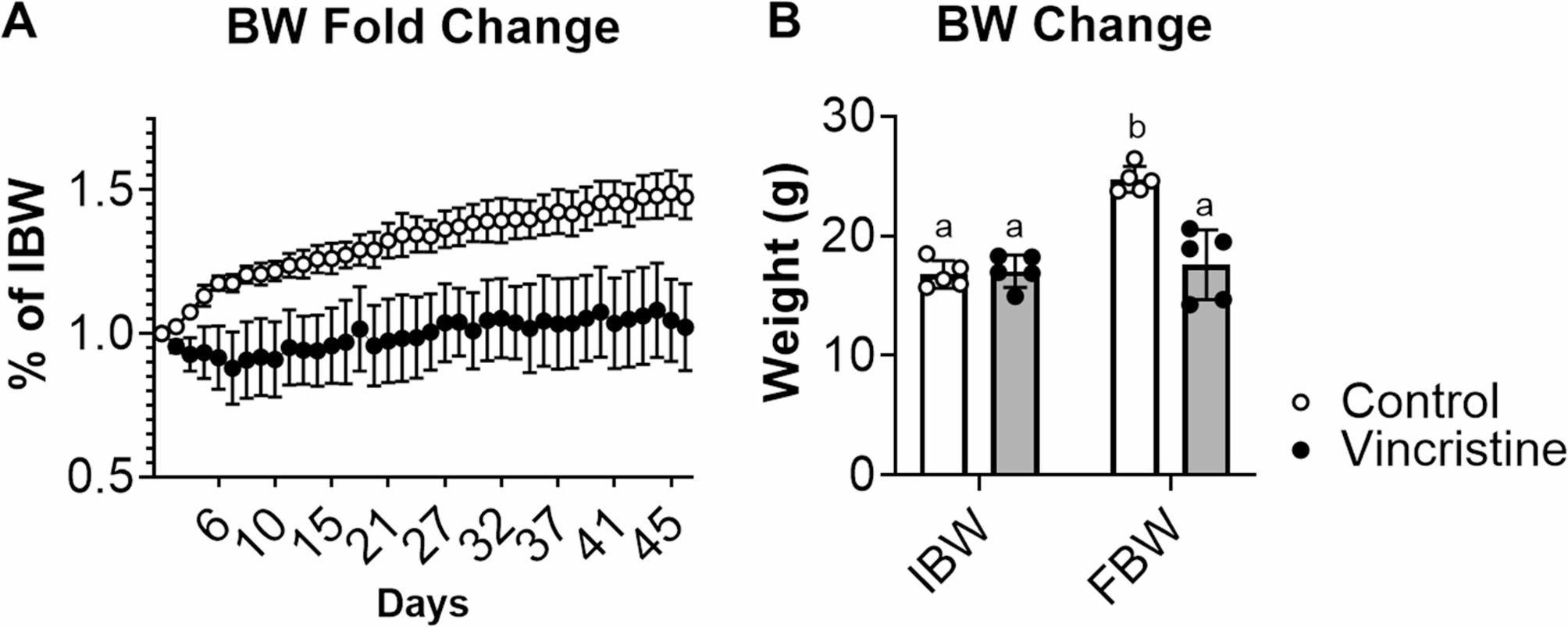
Vincristine treatment mitigates gains in body weight. **A** Body weight curves, expressed as percentage change vs. initial body weight (IBW). **B** Body weight (BW) changes displayed as initial body weight (IBW) to final body weight (FBW), expressed in grams (g). Data expressed as mean ± SD. Different letters denote significant differences: *p*<0.05. Control (*n*=5); Vincristine (*n*=5)

**Fig. 3:**
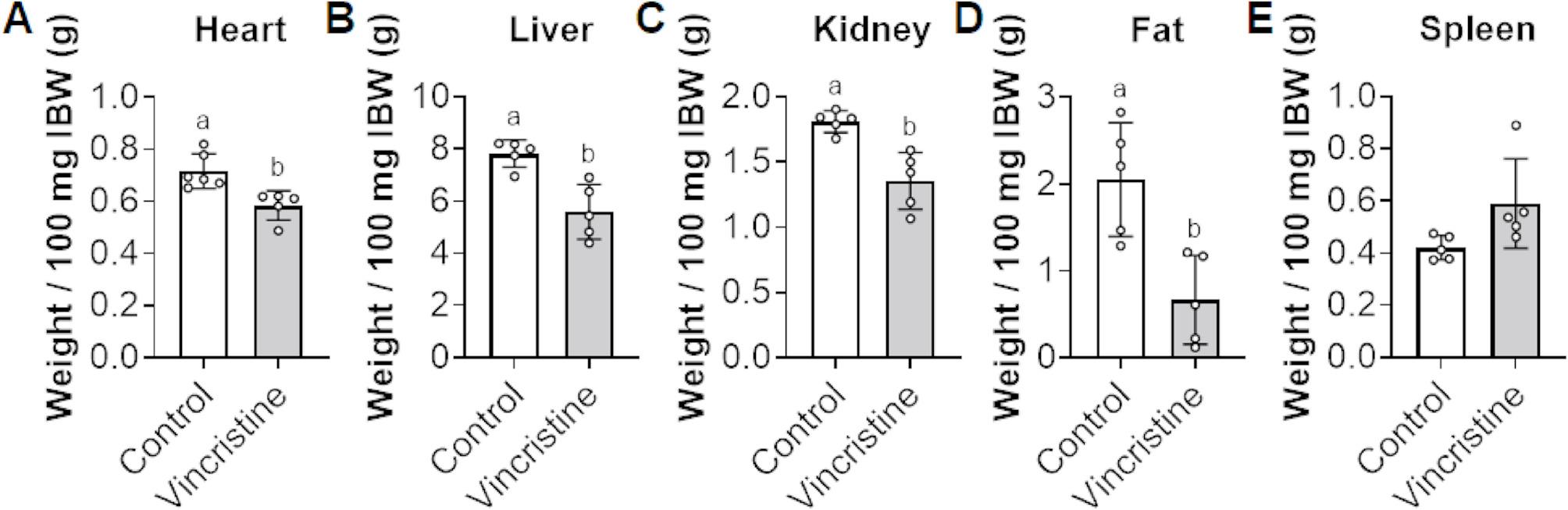
Vincristine treatments reduce heart, liver, kidney, and fat masses – with no effect on spleen. **A** Heart, **B** Liver, **C** Kidney, **D** Fat, and **E** Spleen were normalized to initial body weight (IBW) and reported as weight/100 mg of IBW. Data expressed as mean ± SD. Different letters denote significant differences: *p*<0.05. Control (*n*=5); Vincristine (*n*=5)

To assess the systemic impact of vincristine on musculoskeletal development, 4-week-old male C57BL/6J mice were administered vincristine (1.5 mg/kg, twice weekly) over a 5-week period (Fig. 2). As shown by the body weight curves in Fig. 2A, vincristine attenuated growth compared to the control animals. While no significant difference in initial body weight (IBW) between control and vincristine-treated mice were observed (Control IBW: 16.8 ± 1.1 g; Vincristine IBW: 17.1 ± 1.4 g; Fig. 2B), following 5 weeks of treatment the control mice exhibited a 47% increase in body weight (Control IBW: 16.8 ± 1.1 g; Control FBW (Final Body Weight): 24.7 ± 1.1 g; *p* < 0.05) whereas the vincristine-treated mice showed no significant weight gain (Vincristine FBW: 17.1 ± 1.4 g; Vincristine FBW: 17.6 ± 2.9 g; *p* > 0.05) (Fig. 2B). This resulted in a 29% difference in final body weight between the vincristine-treated group compared to controls (Control FBW: 24.7 ± 1.1 g; Vincristine FBW: 17.6 ± 2.9 g; *p* < 0.05) (Fig. 2B). Notably, the vincristine-treated mice also exhibited diminished organ weights, reported both as values normalized to IBW (Fig. 3) and as raw measurements (Figure S1). Notably, reductions were observed in the heart (-19%, *p* < 0.05 vs. control), liver (-29%, *p* < 0.05 vs. control), kidney (-25%, *p* < 0.05 vs. control), and gonadal fat (-67%, *p* < 0.05 vs. control) (Fig. 3A-D). In contrast, spleen weight remained unchanged (Fig. 3E). These results suggest that vincristine not only stunts overall growth but also reduces the mass of metabolically and hormonally active organs.

### Vincristine reduces skeletal muscle mass and ex vivo muscle force

**Fig. 4:**
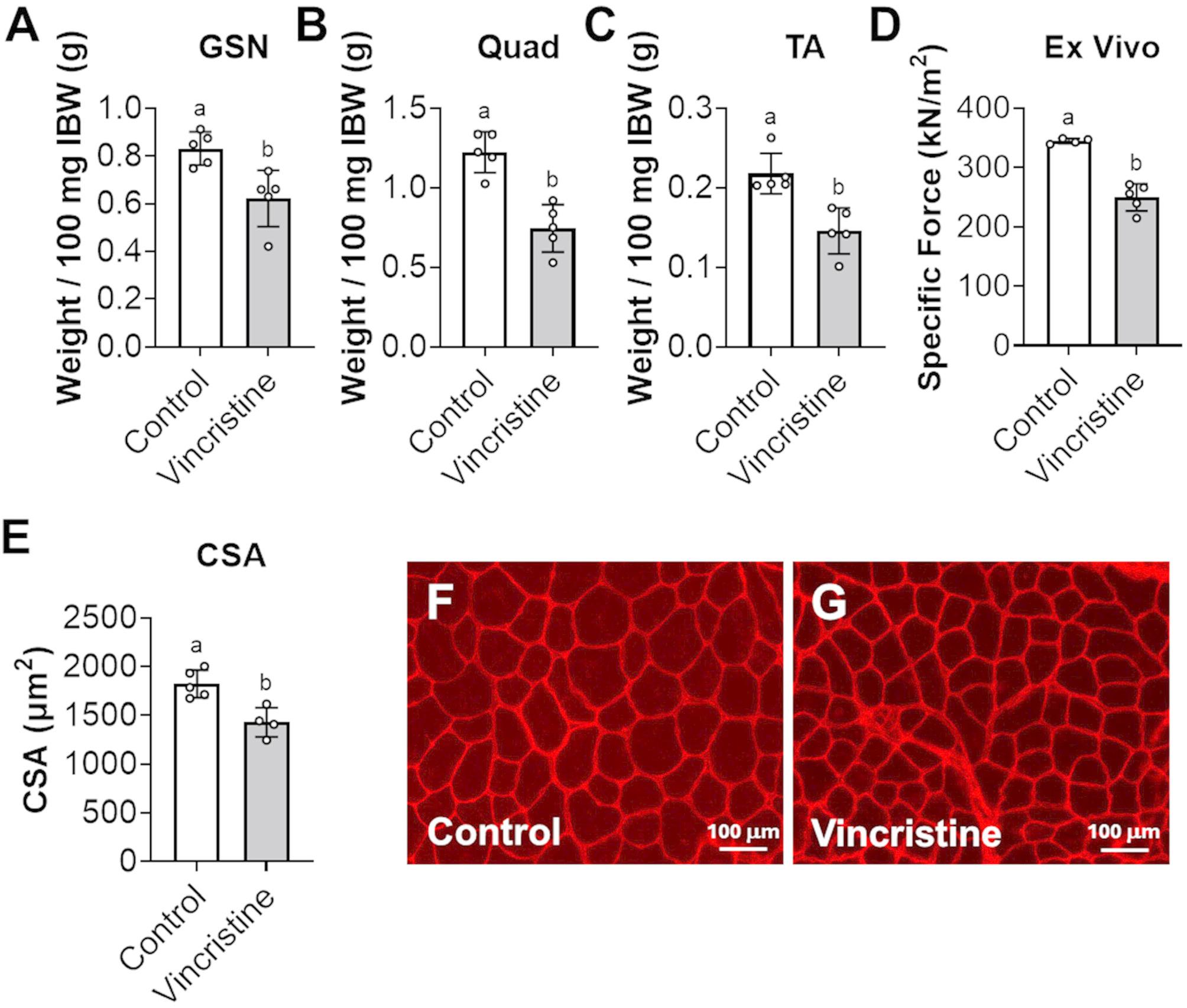
Vincristine treatments reduce skeletal muscle weight, and ex vivo EDL force and muscle cross sectional area. **A** Gastrocnemius (GSN), **B** quadriceps (Quad), **C** tibialis anterior (TA) muscle weights were normalized to initial body weight (IBW) and expressed as weight/100 mg IBW. **D** EDL peak ex vivo force measured at 150Hz. Ex vivo force expressed as kN/m2. **E** Average cross-sectional area (CSA) of myofibers was expressed as µm2. **F**, **G** Representative image of tibialis anterior muscle cross sections. Data expressed as mean ± SD. Different letters denote significant differences: *p*<0.05. Control (*n*=5); Vincristine (*n*=5)

Vincristine treatment significantly impaired skeletal muscle development in pediatric mice. Compared to controls, the vincristine-treated animals exhibited reductions in quadriceps (-39%, *p* < 0.05), gastrocnemius (-25%, *p* < 0.05), and tibialis anterior muscles mass (-33%, *p* < 0.05) (Fig. 4A-C). Functionally, vincristine also compromised muscle performance. Ex vivo force measurements of EDL muscles showed a 28% decrease in maximal force production in the vincristine group compared to controls (*p* < 0.05) (Fig. 4D). Lastly, histological analysis of tibialis anterior cross-sections revealed a 22% reduction in average muscle fiber cross-sectional area (CSA) in the vincristine treated mice (Control: 1821 ± 141 µm^2^; Vincristine: 1429 ± 150 µm^2^; *p* < 0.05) (Fig. 4E-G).

### Vincristine causes a shift towards more glycolytic fibers

**Fig. 5:**
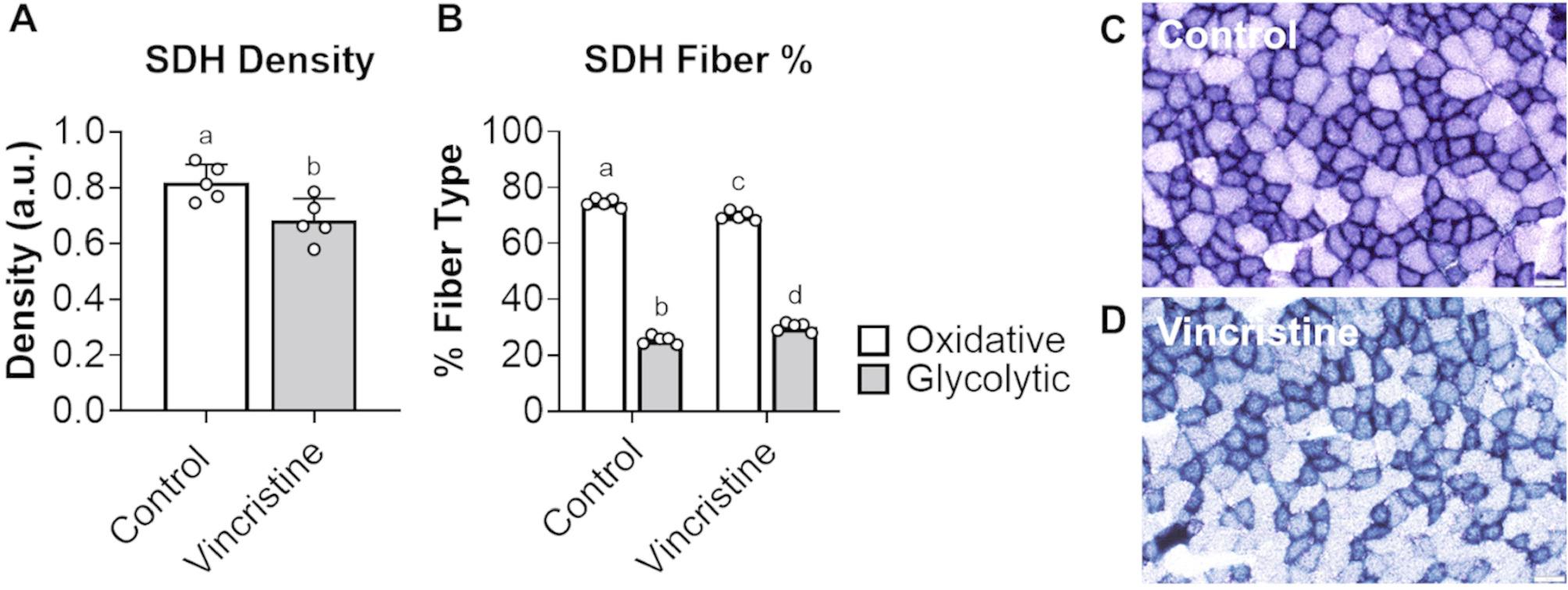
Vincristine treatments promote an oxidative-to-glycolytic shift in muscle fiber composition. **A** Optical density of SDH staining images, higher optical density indicative of greater SDH concentrations. **B** Percentage of oxidative vs. glycolytic muscle fibers in control and vincristine-treated animals. **C**, **D** Representative images of tibialis anterior cross sections in control and vincristine-treated mice. Data expressed as mean ± SD. Different letters denote significant differences: *p*<0.05. Control (*n*=5); Vincristine (*n*=5)

To determine the effects on skeletal muscle fiber composition, succinate dehydrogenase (SDH) staining was performed on tibialis anterior cross-sections. Vincristine treatment resulted in a noticeable reduction in SDH staining intensity (Fig. 5A), indicating an overall decrease in mitochondrial function and supporting a shift in fiber composition from an oxidative (Control: 74.5%; Vincristine: 69.9%; *p* < 0.001) toward a more glycolytic (Control: 25.5%; Vincristine: 30.1%; *p* < 0.001) phenotype (Fig. 5B-D). These findings suggest that vincristine not only reduces muscle mass and function but also alters the metabolic profile of skeletal muscle, favoring a less oxidative, more glycolytic phenotype.

### Vincristine upregulates markers associated with skeletal muscle atrophy

**Fig. 6:**
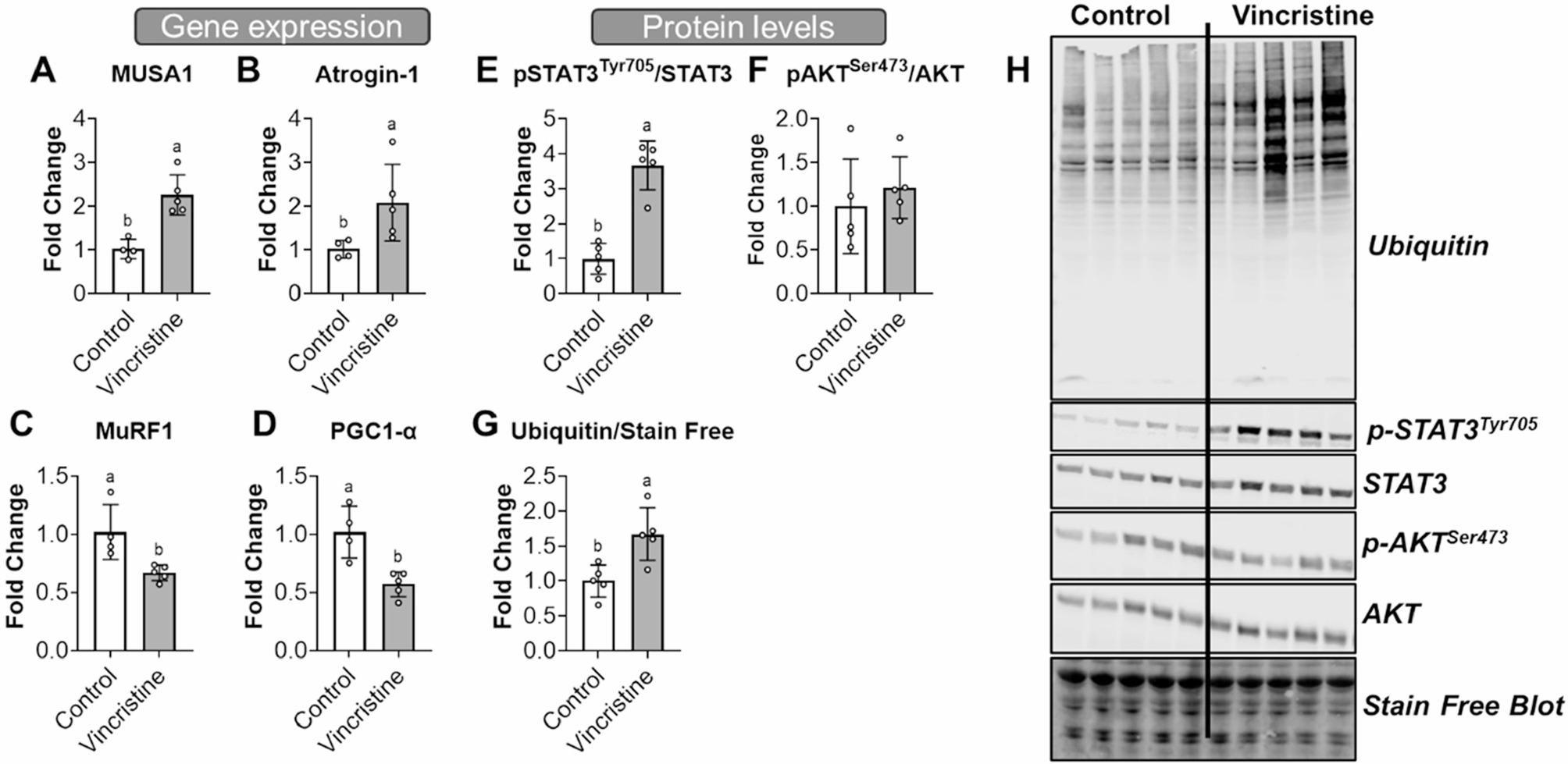
Vincristine yields divergent muscle-specific ubiquitin ligases gene expression, downregulates PGC1-⍺ gene expression and increases protein degradation and phosphorylation of STAT3 in mouse skeletal muscle. Gene expression (via qPCR) of **A** MUSA1, **B** Atrogin-1, **C** MuRF1, and **D** PGC1-⍺. Protein levels (assessed via Western blotting) for **E** phospho-STAT3Tyr705, **F** phospho-AKTSer473 and **G** total ubiquitin. **H** Representative Western blotting in quadriceps muscle from control and vincristine-treated animals. Expression levels reported as fold change vs. control. Data expressed as mean ± SD. Different letters denote significant differences: *p*<0.05. Control (*n*=5); Vincristine (*n*=5)

To investigate the molecular mechanisms underlying vincristine-induced muscle atrophy, we analyzed mRNA and protein expression in quadriceps muscle tissue. Interestingly, we observed a differential expression of muscle-specific E3 ubiquitin ligases, with a significant upregulation of MUSA1 (+ 122%, *p* < 0.05) and Atrogin-1 (+ 105%, *p* < 0.05), and a downregulation of MuRF1 (+ 34%, *p* < 0.05) vs. controls (Fig. 6A-C). Additionally, PGC-1α, a key regulator of mitochondrial biogenesis and oxidative metabolism, was significantly decreased in the muscle of vincristine treated mice (+ 44%, *p* < 0.05 vs. control), further suggesting impaired mitochondrial function (Fig. 6D). Furthermore, phosphorylated STAT3 levels were elevated by 267% compared to controls (*p* < 0.05), indicating activation of pro-atrophic signaling pathways (Fig. 6E), whereas no changes in the phosphorylation of AKT^Ser473^ (*p* < 0.05) were noted (Fig. 6F). Furthermore, protein degradation also appeared to be enhanced upon vincristine treatment, as evidenced by a 67% increase in total protein ubiquitination (*p* < 0.05) vs. controls (Fig. 6G). These findings collectively suggest that vincristine promotes skeletal muscle atrophy through transcriptional and post-translational mechanisms involving increased protein degradation and altered mitochondrial signaling.

### Vincristine impairs trabecular and cortical bone architecture

**Fig. 7:**
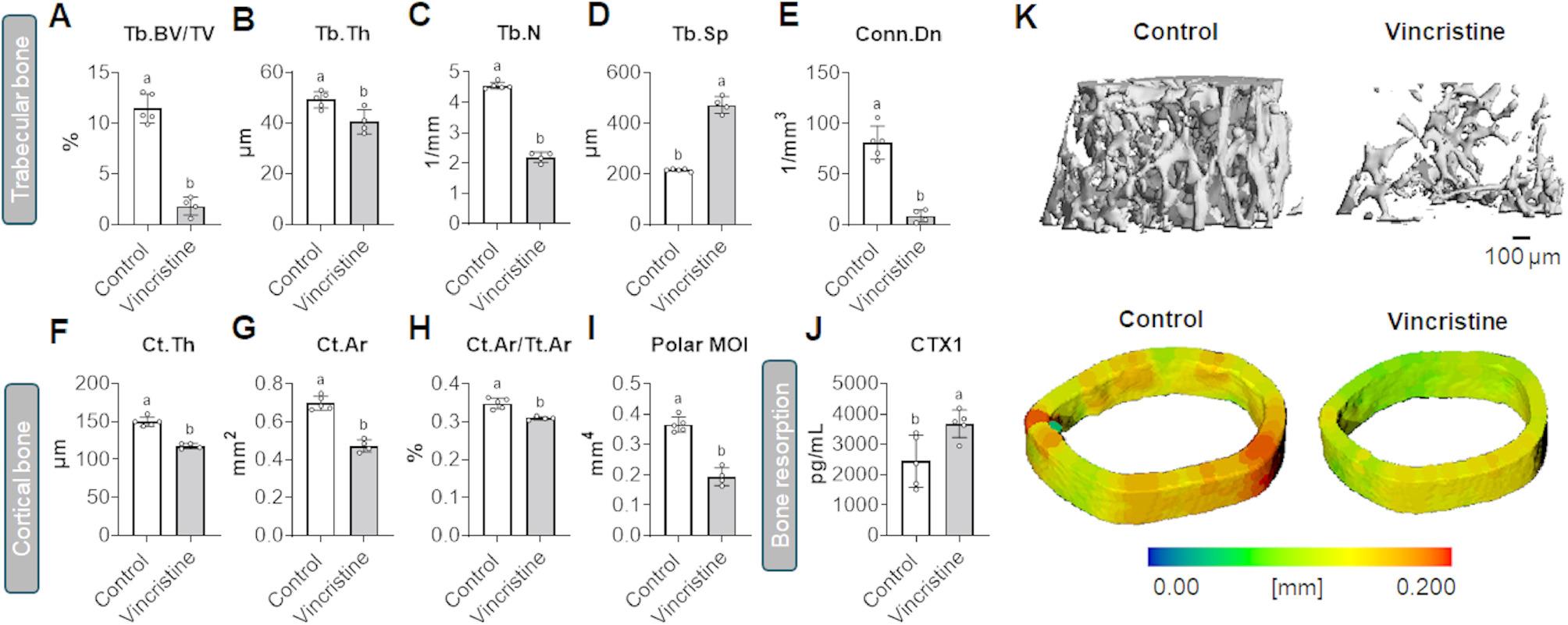
Vincristine affects trabecular, cortical bone and increases a plasma marker associated with bone resorption (CTX-1). **A** Quantification of trabecular bone volume fraction (Tb.BV/TV; %), **B** trabecular thickness (Tb.Th; μm), **C** trabecular number (Tb.N; μm), **D** trabecular spacing (Tb.Sp; μm), **E** connectivity density (Conn.Dn; 1/mm3), **F** cortical thickness (Ct.Th: mm), **G** cortical area (Ct.Ar; mm2), **H** fraction of cortical bone relative to total bone (Ct.Ar/Tt.Ar; %) in the femur of mice. **I** resistance to force (Polar MOI; J:mm4). **J** Plasma C-terminal telopeptide of type I collagen (CTX-1; pg/mL). **K** Representative 3-D images of trabecular bone and cortical bone. Data reported as means ± SD. Different letters denote significant differences: *p*<0.05. Control (*n*=5); Vincristine (*n*=5)

Our recent studies indicate that different chemotherapeutics drive significant losses of bone mass, consistent with changes in bone volume fraction (BV/TV, indicative of the volume of mineralized bone per unit volume of sample), trabecular thickness (Tb.Th, i.e., the average thickness of the trabeculae), trabecular number (Tb.N, representing the number of trabeculae per unit of length), connectivity density (Conn.Dn, indicating the trabecular connections per unit of volume) and trabecular spacing (Tb.Sp, a measure of the mean distance between the borders of the segmented trabeculae). Therefore, in this study we assessed the impact of vincristine on bone microarchitecture by performing micro-computed tomography (µCT) analysis of femurs from treated and control mice. Vincristine treatment resulted in substantial trabecular bone loss, as evidenced by decreased BV/TV (-84%, *p* < 0.05), Tb.Th (-18%, *p* < 0.05), Tb.N (-52%, *p* < 0.05), and Conn.Dn (-89%, *p* < 0.05), and by augmented Tb.Sp (+ 120%, *p* < 0.05) (Fig. 7A-E). In addition to trabecular deterioration, cortical bone structure was also significantly compromised, as suggested by decreased cortical thickness (Ct.Th; -21%, *p* < 0.05), cortical area (Ct.Ar.; -32%, *p* < 0.05) and cortical bone fraction of total bone (Ct.Ar/Tt.Ar; -11%, *p* < 0.05) (Fig. 7F-H). Interestingly, we also observed a decrease in the femur’s resistance to torsion (Polar MOI; -53%, *p* < 0.05) (Fig. 7I). Lastly, to evaluate bone resorption, plasma C-terminal telopeptide of type I collagen (CTX-1) was measured. Vincristine-treated mice exhibited a significant increase in CTX-1, indicating elevated bone resorption (+ 51%, *p* < 0.05) (Fig. 7J). Lastly, there was significant difference between femur lengths between groups (Control: 14.8 ± 0.1; Vincristine: 13.7 ± 0.8 mm; *p* < 0.05), therefore also suggesting an overall effect of vincristine on bone growth.

## Discussion

Given the limited research on chemotherapy-induced musculoskeletal toxicity in young animals, we sought to investigate the immediate effects of vincristine, a chemotherapeutic commonly prescribed in pediatric oncology, on skeletal muscle health in juvenile mice. Vincristine functions as a mitotic poison, disrupting microtubule formation and preventing chromosome segregation during mitosis, ultimately leading to mitotic arrest and apoptosis [12–14]. Its distinct mechanism of action relative to other chemotherapeutics provides an opportunity to explore whether this specific class of chemotherapeutics elicits unique musculoskeletal responses during early developmental stages.

To investigate this point, we administered vincristine to 4-week-old mice over a 5-week period. Vincristine significantly delayed body growth compared to controls, resulting in a 29% reduction in final body weight. This effects is consistent with previous findings, where a milder vincristine regimen (0.75 mg/kg, twice weekly for 4 weeks starting at 3.5 weeks of age) produced a 39% difference in FBW between groups [19]. Notably, both vincristine protocols induced greater growth suppression than the 15% FBW reduction reported with Folfiri in pediatric models [8]. In contrast, cisplatin administration in adult mice typically results in substantial weight loss, with reductions ranging from 12% to 30% of initial body weight [7, 20, 21], while its impact in pediatric subjects remains undetermined. Interestingly, while chemotherapy in adult models often leads to overt weight loss [7, 22], our pediatric studies [8], including the present one, showed modest weight gains (+ 10% with Folfiri and + 3% with Vincristine), suggesting a different physiological response. These findings underscore the complexity of characterizing chemotherapy-induced musculoskeletal toxicity in pediatric populations. Rather than aligning with the traditional cachexia paradigm, the phenotype observed here is marked by skeletal muscle mass loss accompanied by minimal overall weight gain during a critical developmental window. This presentation is more accurately described as *“failure to thrive”* or *“growth faltering”*. Recognizing this distinction is essential for understanding and addressing chemotherapy-induced growth impairments in pediatric settings.

In adult rodent models, chemotherapy consistently induces skeletal muscle atrophy and functional decline [7, 20–22]. Extending these findings to pediatric models, our recent work demonstrated that Folfiri similarly reduces both skeletal muscle mass and ex vivo muscle force in young animals, with some effects persisting weeks after treatment cessation [8]. In the present study, we showed that vincristine similarly impaired skeletal muscle development and function in pediatric mice, thereby reinforcing clinical observations of reduced lean muscle mass and muscle weakness in pediatric cancer patients undergoing chemotherapy treatments [23, 24]. Our observations are also consistent with previous research showing a dose-dependent reduction in ex vivo EDL muscle function after a single vincristine treatment in adult rats [25].

At the molecular level, skeletal muscle atrophy, whether due to disease or aging, is often associated with activation of the ubiquitin-proteasome system (UPS), including increased expression of muscle-specific E3 ubiquitin ligases such as MUSA1, MuRF-1, and Atrogin-1, as well as elevated levels of protein ubiquitination [26–29]. In our study, vincristine-treated animals exhibited increased muscle protein ubiquitination, suggesting enhanced protein degradation. However, the expression of E3 ubiquitin ligases was not uniform. While MUSA1 was upregulated, MuRF-1 was downregulated, and Atrogin-1 remained unchanged. These findings only partially align with previous studies showing that chemotherapy can upregulate E3 ligases such as Atrogin-1 and MuRF-1 [20, 21]. Altogether, these results suggest that chemotherapy-induced UPS activation may be agent-specific, and not all E3 ligases respond uniformly across treatments.

Mitochondrial dysfunction is another well-established contributor to skeletal muscle atrophy [30]. Our previous work has demonstrated that chemotherapy alone impairs mitochondrial homeostasis [6–8], and that preservation of the mitochondrial pool can drastically reduce chemotherapy-related muscle toxicities [7]. For example, cisplatin administration in mice led to downregulation of key mitochondrial markers, including PGC-1α, OPA1, VDAC, Cytochrome C, and COXIV, independent of age and sex [7]. Similarly, Folfiri treatment persistently disrupted oxidative phosphorylation, altered lipoprotein metabolism, and increased systemic inflammation, with mitochondrial defects observed even after a 4-week washout period following chemotherapy treatment in the pediatric mice [8]. In this study, vincristine reduced PGC-1α gene expression and diminished the oxidative metabolism in skeletal muscle, as also suggested by the shift in fiber composition, further supporting the notion that chemotherapy compromises mitochondrial function across different agents and age groups. Although the precise mechanisms by which vincristine induces mitochondrial dysfunction in skeletal muscle remain to be elucidated, previous studies have demonstrated its deleterious impact on neuronal mitochondria, particularly in the context of peripheral neuropathy. Notably, vincristine has been shown to disrupt calcium homeostasis by impairing mitochondrial calcium transport, thereby compromising both uptake and efflux processes and ultimately altering mitochondrial function [31, 32]. While these specific derangements were not directly examined in the present study, they underscore a fundamental vulnerability of mitochondrial integrity to chemotherapeutic stress.

Our findings showed that chemotherapy administration during early postnatal development leads to widespread musculoskeletal toxicity including significant losses of skeletal muscle mass and contractile function and drastic changes in bone integrity. Bone loss is a particularly critical clinical concern due to its association with osteoporosis, delayed growth and height loss, functional impairment, increased fracture risk and reduced quality of life. Consistent with our previous work in adult [20] and pediatric [8] mouse models, vincristine treatment resulted in marked reductions in trabecular bone volume, number, and connectivity density, alongside increased trabecular spacing. Cortical bone was similarly affected, with decreased thickness and potentially compromised mechanical strength, as indicated by reduced polar moment of inertia (MOI). To provide insight on the potential mechanisms involved in bone resorption following vincristine treatment, we measured plasma levels of CTX-1, a marker of bone resorption commonly elevated in osteoporosis and cancer cachexia [33–35]. Vincristine-treated mice showed significantly higher plasma CTX-1 levels, supporting the conclusion that vincristine promotes bone degradation. Our findings align with clinical observations that childhood cancer survivors often exhibit reduced bone mineral density into adulthood, underscoring the long-term skeletal consequences of a cancer diagnosis [36].

This study builds upon our recent work investigating the immediate and persistent effects of Folfiri treatment in pediatric mouse models [8] and describes new important observations. However, a key limitation of our analysis is the absence of a post-treatment washout period, which precludes assessment of long-term recovery or persistence of musculoskeletal deficits. Additionally, and similar to our previous research using pediatric mouse models [8, 9], the use of young mice undergoing rapid growth introduces potential confounding variables. While control mice exhibited a 47% increase in body weight over the study period, the vincristine-treated mice showed no significant weight gain. Despite this, we detected elevated markers of protein degradation (e.g., ubiquitin) and skeletal muscle atrophy (e.g., phosphorylated STAT3^Tyr705^), which are typically associated with cachexia in adult models. Interestingly, vincristine treatment resulted in non-canonical expression patterns of E3 muscle-specific ubiquitin ligases. This discordant regulation, along with reduced PGC-1α expression, suggests that pediatric muscle may engage distinct signaling pathways in response to chemotherapy. Supporting this, our recent work in a pediatric glioblastoma model also revealed downregulation of E3 ligase gene expression [9], reinforcing the idea that pediatric cancer and chemotherapy may elicit unique molecular responses. Another limitation of this study is the use of chemotherapy in the absence of cancer, which does not fully replicate the clinical scenario. Future investigations should incorporate pediatric-relevant tumor models to better reflect the complex interactions between cancer, chemotherapy, and musculoskeletal development.

## Conclusion

Our data demonstrates that vincristine exerts broad and multifaceted detrimental effects on musculoskeletal development during early life. In vitro, vincristine promoted myotube atrophy, whereas in vivo, it impaired body and muscle growth, reduced muscle fiber cross-sectional area, and induced a shift from oxidative to glycolytic in muscle fiber composition. Additionally, vincristine significantly compromised both trabecular and cortical bone architecture and elevated markers of bone resorption. At the molecular level, vincristine upregulated markers of skeletal muscle atrophy, increased protein ubiquitination, and downregulated PGC-1α, a key regulator of mitochondrial biogenesis. Together, these findings underscore the vulnerability of the developing musculoskeletal system to chemotherapeutic stress and highlight the need for targeted strategies to mitigate these adverse effects in pediatric cancer patients.

## Supplementary Information

Supplementary Material 1.

## Data Availability

Data and materials will be available upon request.

## Abbreviations

ANOVA: Analysis of Variance
BCA: Bicinchoninic Acid (protein assay method)
BV/TV: Bone Volume / Total Volume
CON: Control (used to denote control group in experiments)
CO₂: Carbon Dioxide
CSA: Cross-Sectional Area
CT: Cycle Threshold (in qPCR); also used in µCT context
CTX-1: C-terminal Telopeptide of Type I Collagen
DMEM: Dulbecco’s Modified Eagle Medium
EDL: Extensor Digitorum Longus (muscle)
ELISA: Enzyme-Linked Immunosorbent Assay
MF-20: Myosin Heavy Chain Antibody (clone MF-20)
MOI: Moment of Inertia (Polar MOI in bone analysis)
MUSA1: Muscle Ubiquitin Ligase of SCF Complex in Atrophy-1
MuRF-1: Muscle RING-Finger Protein-1
NP-40: Nonidet P-40
NaCl: Sodium Chloride
PBS: Phosphate-Buffered Saline
PCR: Polymerase Chain Reaction
PFA: Paraformaldehyde
PGC-1α: Peroxisome Proliferator-Activated Receptor Gamma Coactivator 1-alpha
RIPA: Radioimmunoprecipitation Assay
RNA: Ribonucleic Acid
SD: Standard Deviation
SDH: Succinate Dehydrogenase
SDS: Sodium Dodecyl Sulfate
STAT3: Signal Transducer and Activator of Transcription 3
TBP: TATA-Binding Protein
TBS: Tris-Buffered Saline
TBS-T: Tris-Buffered Saline with Tween 20
Tb.N: Trabecular Number
Tb.Sp: Trabecular Separation
Tb.Th: Trabecular Thickness
Tiss.D: Tissue Mineral Density
UPS: Ubiquitin-Proteasome System
VDAC: Voltage-Dependent Anion Channel
mRNA: Messenger Ribonucleic Acid
qPCR / qRT-PCR: Quantitative Real-Time Polymerase Chain Reaction
µCT: Micro-Computed Tomography
