# Supplementary material for: Vincristine impairs musculoskeletal development in pediatric mice
Source: BMC Cancer. 2025 Nov 18;25:1782. doi: 10.1186/s12885-025-15262-x (PMC12625613; doi:10.1186/s12885-025-15262-x)
Supplement: Supplementary file 1 — Supplementary Material 1. [file 12885_2025_15262_MOESM1_ESM.pptx]

## Slide 1
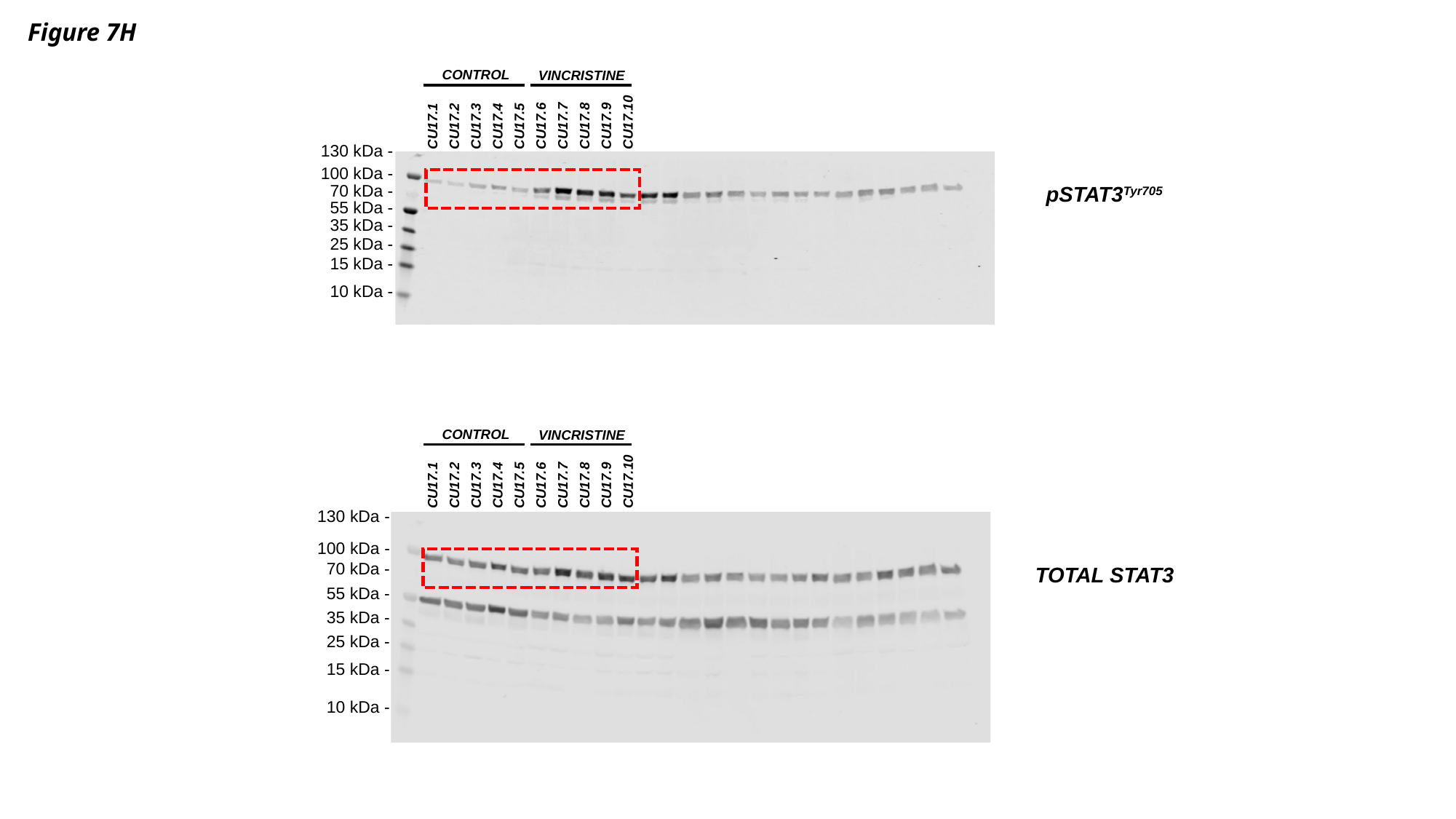

Figure 7H
CONTROL
VINCRISTINE
CU17.1
CU17.2
CU17.3
CU17.4
CU17.5
CU17.6
CU17.7
CU17.8
CU17.9
CU17.10
130 kDa -
100 kDa -
70 kDa -
pSTAT3Tyr705
55 kDa -
35 kDa -
25 kDa -
15 kDa -
10 kDa -
CONTROL
VINCRISTINE
CU17.1
CU17.2
CU17.3
CU17.4
CU17.5
CU17.6
CU17.7
CU17.8
CU17.9
CU17.10
130 kDa -
100 kDa -
70 kDa -
TOTAL STAT3
55 kDa -
35 kDa -
25 kDa -
15 kDa -
10 kDa -

## Slide 2
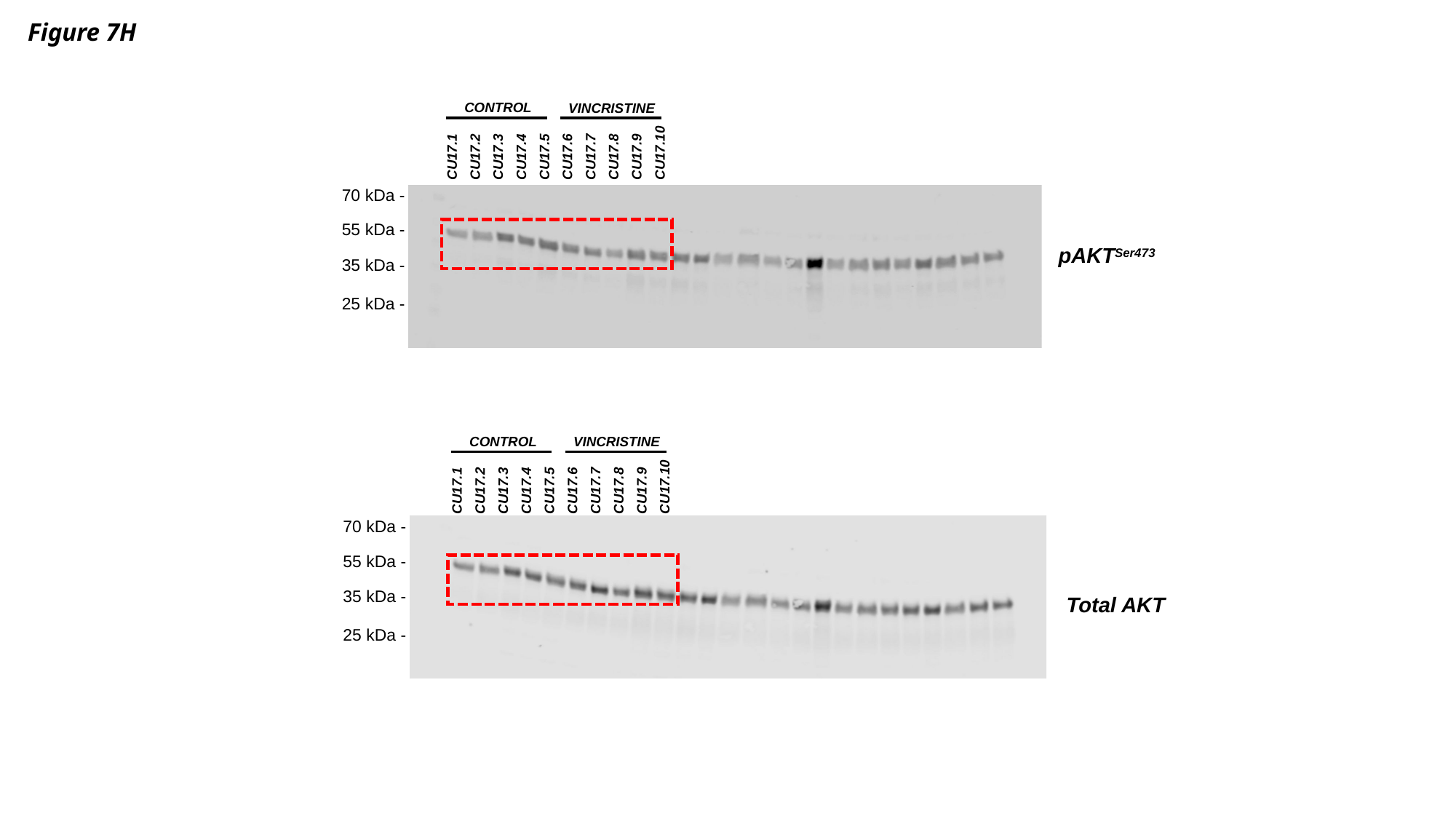

Figure 7H
CONTROL
VINCRISTINE
CU17.1
CU17.2
CU17.3
CU17.4
CU17.5
CU17.6
CU17.7
CU17.8
CU17.9
CU17.10
70 kDa -
55 kDa -
pAKTSer473
35 kDa -
25 kDa -
CONTROL
VINCRISTINE
CU17.1
CU17.2
CU17.3
CU17.4
CU17.5
CU17.6
CU17.7
CU17.8
CU17.9
CU17.10
70 kDa -
55 kDa -
35 kDa -
Total AKT
25 kDa -

## Slide 3
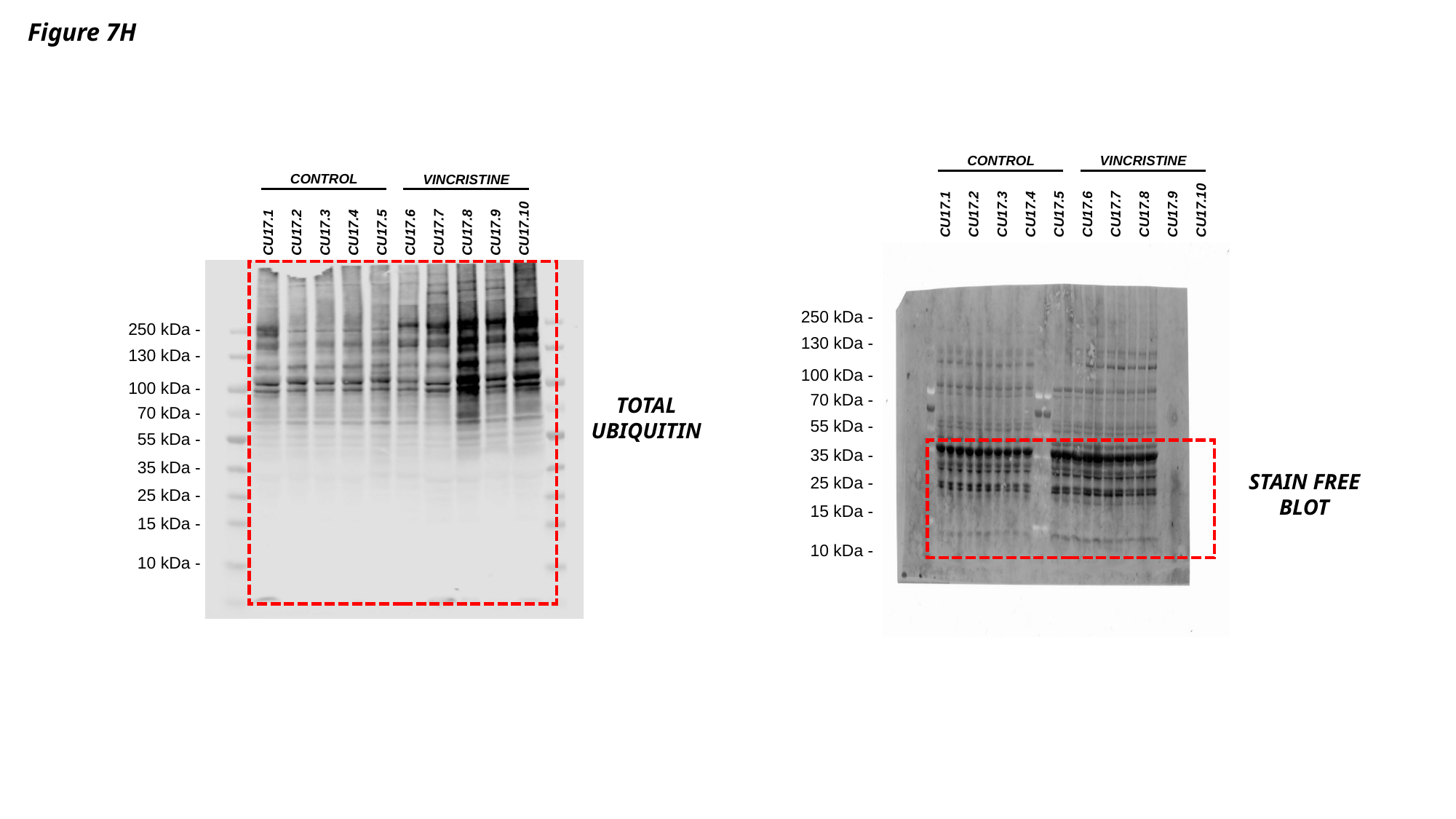

Figure 7H
CONTROL
VINCRISTINE
CU17.1
CU17.2
CU17.3
CU17.4
CU17.5
CU17.6
CU17.7
CU17.8
CU17.9
CU17.10
250 kDa -
130 kDa -
100 kDa -
70 kDa -
55 kDa -
35 kDa -
STAIN FREE BLOT
25 kDa -
15 kDa -
10 kDa -
CONTROL
VINCRISTINE
CU17.1
CU17.2
CU17.3
CU17.4
CU17.5
CU17.6
CU17.7
CU17.8
CU17.9
CU17.10
250 kDa -
130 kDa -
100 kDa -
TOTAL UBIQUITIN
70 kDa -
55 kDa -
35 kDa -
25 kDa -
15 kDa -
10 kDa -

## Slide 4
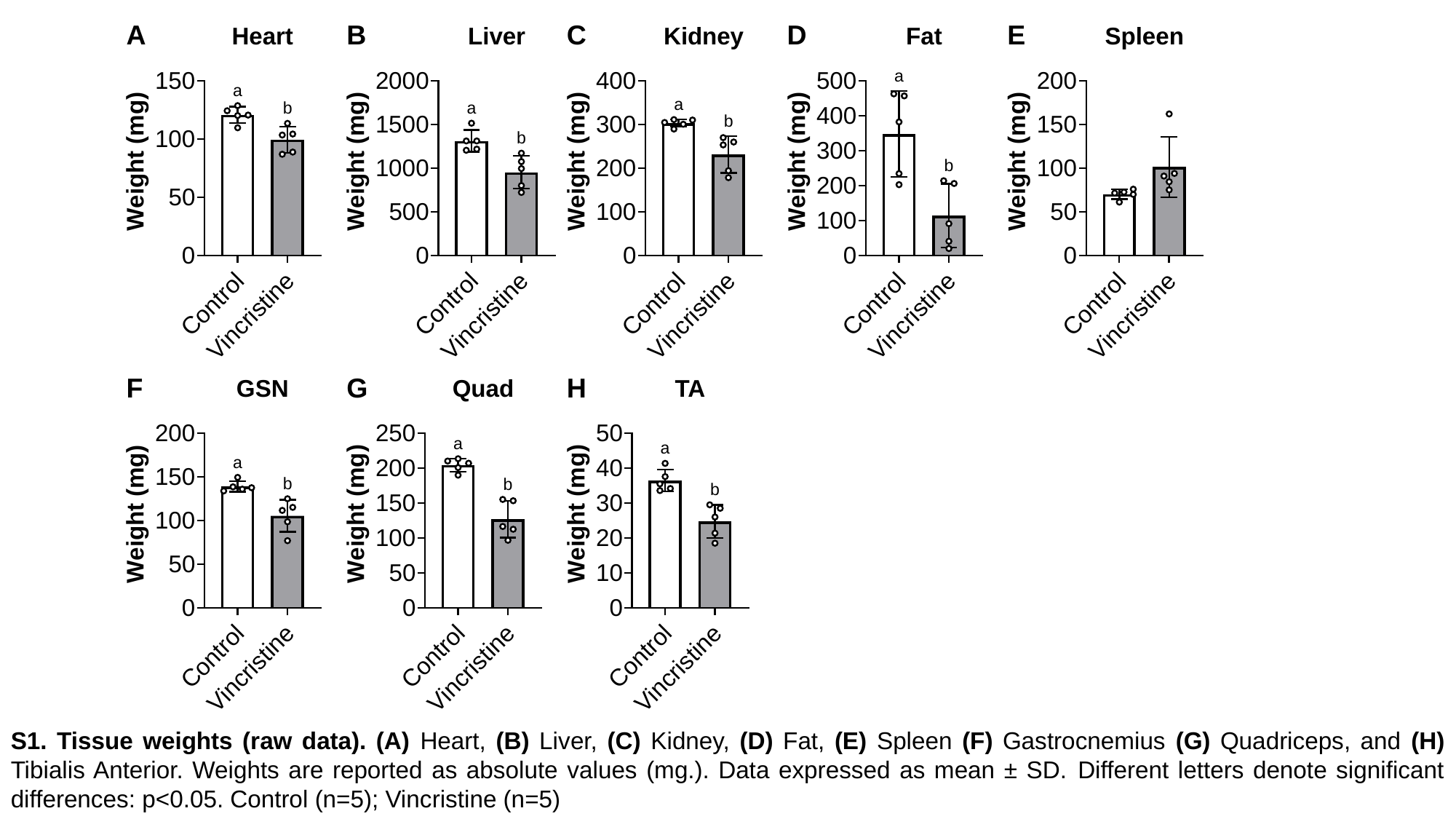

A
B
C
D
E
F
G
H
S1. Tissue weights (raw data). (A) Heart, (B) Liver, (C) Kidney, (D) Fat, (E) Spleen (F) Gastrocnemius (G) Quadriceps, and (H) Tibialis Anterior. Weights are reported as absolute values (mg.). Data expressed as mean ± SD. Different letters denote significant differences: p<0.05. Control (n=5); Vincristine (n=5)
